# Commentary - Community Involvement and Prevention Science in and with Communities – Where do we go next?

**DOI:** 10.1007/s10935-025-00864-9

**Published:** 2025-06-28

**Authors:** Jeremy Segrott, Ina Koning, Boris Chapoton

**Affiliations:** 1 Centre for Trials Research, DECIPHer Centre, Cardiff University, Wales, UK; 2 Clinical Child and Family Studies, Vrije Universiteit, Amsterdam, Netherlands; 3 Université Jean Monnet, Saint-Etienne, CoActiS UR4161 France

**Keywords:** Prevention Science, Communities, Community involvement, Public involvement, Participatory research

## Abstract

This commentary piece reflects on the 2024 EUSPR Conference and its theme of ‘Prevention in and with communities’. We discuss the challenges that Prevention Science needs to address as it develops community involvement in its work. After briefly summarising some of the key definitions and boundaries of community involvement, we consider three key challenges which were highlighted during the Cremona conference. The first concerns the importance of building skills and capacity for community involvement and the role of organisations such as the EUSPR. Second, we explore the challenges of balancing involvement of the community and safeguarding evidence-based-practice principles (EBP), and potential strategies to achieve this. Community involvement need not be in opposition to research evidence or the principles of EBP. Third, the value of assessing the impact and quality of community involvement is discussed. We look at the need for researchers to report on the design and outcomes of community involvement, and the imperative to avoid causing harm such as excluding certain individuals/groups. In the concluding section of the commentary, we answer the question of ‘Where do we go next?’ by highlighting some specific steps in which the EUSPR (and other Prevention Science societies) have an important contribution to make. These include training and capacity building, knowledge exchange on the implementation of community involvement within research projects and developing dialogues with the public whose communities the EUSPR conference takes place within.

## A Week in Cremona

The 2024 15th EUSPR conference in Cremona, Italy, took as its theme ‘Prevention in and with communities’. Presentations, posters, and informal conversations produced valuable insights, including lessons learned from the field, and were often future- and action-oriented. They mapped out how Prevention Science might develop community involvement. This encompassed the benefits of doing so, how we integrate the lived experiences of communities with the principles of evidence-based practice, and the skills and infrastructures needed to support high quality community involvement.

EUSPR’s website described the conference as aiming to:

*… connect research with practical needs of communities*,* discuss pros and cons of different approaches to prevention in and with community settings*,* as well as opportunities and challenges of developing*,* implementing and evaluating evidence-based community-level prevention programmes and systems.* (EUSPR, 2024)

Inspired by what we learnt at the conference, this commentary piece discusses the challenges that Prevention Science needs to address when working in and with communities, whilst noting that all challenges offer opportunities for developing our science. The specific focus of our commentary paper is on Community Involvement – which we conceptualise as being involved in the design and conduct of research. Although we recognise the rich experiences of, and the many challenges faced by policymakers and practitioners when working in and with communities, they are not discussed in this paper, which is concerned with the relationship between researchers and communities.

We do not claim to have all the answers, or even all the questions. This paper is not a comprehensive account of the conference presentations which touched upon community involvement. Our aim is to stimulate discussion and collaboration, and to think about where, as a community of Prevention scientists and practitioners, we might go next.

At the outset it is also important to acknowledge the situated and partial viewpoints which inform our conceptualisations and framing. They stem from our work as academic researchers based in universities, who strive to work with and in communities. But we acknowledge that there are many approaches to undertaking Prevention in communities and multiple perspectives. Thus, when we discuss the ways in which researchers may partner with communities this is not meant to indicate a single way of working but is a product of our own position and experiences. Public involvement – including with communities, has informed each of our respective research careers and practice to date. Segrott (Wales, UK) has worked with advisory groups of young people and parents to design and evaluate family and school-based interventions. Koning (Netherlands) has experience of co-developing and co-evaluation of community-based interventions, particularly in relation to adolescents’ alcohol use and parenting. Chapoton (France) has explored how to enhance existing co-creation practices by involving stakeholders in the development of a school-based program targeting the influence of social networking sites, while also expanding its scope to foster community engagement at multiple levels.

Choosing the theme of Community Involvement for the conference frames it as an important and legitimate set of questions which demand our attention. It signals there may be gaps in our knowledge and practice to fill, and questions to ask about where we go next in developing Prevention Science in and with communities.

### Prevention in and with Communities – Definitions and Boundaries

Beginning with boundaries, our focus in this commentary piece is on Community Involvement in research, specifically within the context of Prevention Science. Research in the field of Prevention comprises the systematic investigation of population needs, evaluation of intervention development, outcomes and implementation, and methodological advances. Although beyond the scope of this paper to explore in detail, it is important to acknowledge that research forms but one – albeit fundamental, aspect of Prevention as a field. Alongside research, Prevention of course encompasses the practice of intervention development and delivery, and the formulation and implementation of policies – at a range of geographical scales. Thus, there has been a long held interest in ‘Community-based Prevention’, which the Committee on Valuing Community-Based Non-Clinical Prevention Programs (2012) define as “involv[ing] members of the affected community in the planning, development, implementation, and evaluation of programs and strategies (Cargo and Mercer, 2008).”

Turning to the specific focus of our paper, we do not seek to provide an exhaustive definition of community involvement in research. There are a range of approaches and terms to describe ways of working with members of the public (Las Nueces et al., 2012), what Aresi et al. (2023) refer to as the ‘participatory paradigm’. These include co-production, co-creation, public involvement, public engagement, and user-centred designs. Likewise, there are many ways to conceptualise and define ‘the public,’ including the population as a whole and specific groups whose needs an intervention is designed to meet. Whilst these concepts are sometimes conceptually distinctive, there is variation in how they are defined, and overlap and inconsistency in the boundaries between them. As Nitsch et al. (2013) suggest, “Many writers have pointed out the sundry definitions and meanings of participation (e.g. Chambers (1995); Morgan (2001) as well as a lack of theoretical underpinnings and conceptual clarity (e.g. Contandriopoulos (2004); Labonte (1997); Marent et al. (2013); Potvin (2007).” Thus, some researchers consider public engagement to be a broad umbrella term for collaboration with the public (Holmes et al., 2019). Others see it as distinct from public involvement - the former being about engaging the public in research, the latter comprising involvement of the public in its design and conduct. For example, the UK Health Research Authority states that “Public involvement is […] different from public engagement, which is when information and knowledge about research is shared with the public” (Health Research Authority, 2024).

By ‘involvement’ we mean activities through which members of the public are involved in the design and conduct of research. We refer to research as the evaluation of prevention interventions (their development, implementation and effectiveness), and research which informs it – e.g. work to identify community needs. Involvement is distinct from being a participant in research. Participants (including those who take part in qualitative interviews) make a valuable contribution to the work that we do but they are not strictly part of the production of the research, even where their views are sought, for example, on the acceptability of an intervention.

Public involvement in the design and conduct of research should be characterised by a two-way exchange of knowledge with researchers. However, it is important to acknowledge that there may still be power imbalances in terms of who initiates a new research study, its overall focus and design, or the actual conduct of the work. Russell (2022) has suggested that community engagement often locates power and authority within the institutions that lead it and can underplay the capacities of communities to organise and define their own priorities. He advocates for a Community Development approach in which “enduring community change happens from the inside out and institutions play a supplementary role in engaging the community’s own capabilities.” Whilst it is beyond the scope of this commentary paper to provide a detailed exploration of community development and building, these are important questions to consider when we (the authors – as researchers) build community involvement in our research. In particular, we take from this discussion that community involvement in research studies should sit within broader collaborative and long term partnerships with communities. Partnerships between researchers and community members might contribute to defining which research projects are needed (and which are not) and do so within the context of ongoing community building efforts. What characterises all of the different forms of involvement/co-working discussed above is the two-way exchange of knowledge, within a process which brings benefits for all those involved (researchers, community members, etc.).

We consider the public to be distinct from policy makers and practitioners (although some community leaders could be seen as activists striving to create the change they want for their community by professionalizing their efforts or becoming politically active). There are of course many ‘publics’, spanning different geographical scales, ages, health needs, and other lived experiences. Community involvement could be thought of as a specific kind of public involvement in which the public are members of a community. Whilst practitioners (e.g. parenting workers, public health coordinators) are part of such communities, we are primarily concerned here with how members of the public can be involved in research. In other words, they are part of – or share similar experiences to, the population for whom an intervention has or will be developed. For example, community involvement in the development of a parenting intervention may focus on seeking the input of parents alongside other forms of involvement work with practitioners and policy makers. Some forms of community involvement are truly participatory (research led by or co-created and co-produced with community members). Other research projects are created and/or led by researchers, with community involvement to some extent happening within a pre-defined set of questions or parameters. These varying approaches all have merit but it is important to be clear and transparent in how we define and operationalise community involvement in our work.

A final point (developed further below) is how to conceptualise prevention interventions in this context. Community involvement might be applied to different research designs (RCTs, other quasi experimental designs, qualitative studies) and to the development of diverse types of interventions (e.g. individual behaviour change, group-based parenting interventions, and community/system wide approaches). Thus, community involvement does not pre-determine intervention type or research design. This also raises the question of what we mean by ‘community.’ We have used terms like ‘involvement’ and ‘public’ (within the community), but what truly defines a community? Is it the way researchers view a population that gravitates around a specific identified problem? Is it the existence of bonds between individuals, shared similarities, a common history, a unified purpose—or all of these at once? Who has the authority to define what makes a community and what does not?

As the 2012 Integrated Framework for Assessing the Value of Community-based Prevention noted, “Community means different things to different people in different context.” However, the definition provided by the authors serves as a useful starting point for this commentary piece:

*… community is defined as any group of people who share geographic space*,* interests*,* goals or history. A community offers a diversity of potential targets for prevention and is often conceived of as an encompassing*,* proximal*,* and comprehensive structure that provides opportunities and resources that shape people’s lifestyle (McIntyre and Ellaway*, 2000).

Given Russell’s critique of community engagement (and the way in which institutions may focus on identifying needs and deficits, rather than existing strengths and capabilities), we think that defining community might involve two related questions, which we can only touch upon briefly here. The first – as in the above quotation, concerns identifying groups of people, and being alive to the ways in which all groups have the potential to exclude as well as connect individuals. Second, communities exist on their own terms and are not waiting to be ‘discovered’ or ‘defined’ by others (including researchers). Whilst researchers with a new research idea may seek to involve communities in its development, there is no shortage of existing ideas within these communities. Where researchers and community members work together over time – sometimes creating formal partnership structures, there is greater diversity in the source of new ideas.

During the Cremona conference, few people clearly identified what constitutes a community. When we speak of the ‘research community,’ for example, do we consider two researchers with different goals within their respective fields to be part of the same community? Would the results of a nuclear experiment directly benefit the research community, including someone working in the arts? Similarly, would research aimed at migrants benefit all migrants equally, or would segmentation be necessary to address specific needs? And once these specificities are addressed, would those classified as part of this community feel a sense of belonging to it?

Many organisations have set out the rationale for public involvement in general (e.g. European Commission, undated; Health Research Authority, 2024; Ligue Contre le Cancer, 2024; Santé Publique France, 2024), and these can be applied to community involvement. One of the main intended benefits is that it strengthens the quality and impact of research. In Prevention Science this relates to maximising the acceptability of a new intervention (e.g. Petelos et al.^2021^) or identifying optimal strategies for participant recruitment. Second, involvement is driven by a commitment to the public’s right to shape research which is about them, or which will affect them (Abelson et al., 2004; Heikkila & Isett, 2007). This rationale is particularly pertinent where public money funds research. Thirdly, community involvement should have mutual benefits for researchers and community members. What members of a community hope to gain through their involvement will differ, but might include a desire to shape research, develop new skills, or the opportunity to connect with others.

### Challenges and Opportunities

Reflecting on what we learnt during the conference and our own work, we highlight three key challenges (and associated opportunities) which need to be addressed to better involve communities in a respectful, fair, and evidence-based manner.

### Skills and Capacity

Prevention Science has a long history of building skills and capacity, including the principles, and application of evidence-based practice. Space precludes a detailed discussion of how we define evidence-based practice. However, Mazzucca et al. (2020) describe evidence-based public health (EBPH) as:

*… an approach to public health practice in which public health practitioners identify*,* implement*,* and evaluate evidence-based interventions (EBIs)*,* including those focused on chronic disease prevention. EBPH is characterized by the use of evidence-based decision-making (EBDM)*,* which is the process of integrating the best available research evidence*,* practitioner expertise*,* and the characteristics*,* needs*,* and preferences of the community (*Brownson et al., 2002; Brownson et al., 1999). *EBDM allows public health practitioners to identify*,* implement*,* and evaluate evidence-based programs and policies that are relevant for their communities (*Brownson et al., 2002).

This definition holds true for Prevention Science. Evidence-based practice is a process in which evidence guides decision making, and the implementation and evaluation of new interventions. The above definition can be critiqued for the way in which practitioners are positioned as the ones which “identify, implement and evaluate evidence-based programs” (albeit with the focus on relevance to “their communities”). Nevertheless it highlights the way in which EBP involves the bringing together of different kinds of evidence, including “the needs, and preferences of the community”.

The principles of building skills and capacity should apply to community involvement. Co-creation is a method – which needs to be learned and refined over time and used to inform intervention development and evaluation. Like all research methods, this requires that training be available so that researchers have the skills they need to undertake high quality community involvement. Training will encompass the concepts and frameworks guiding community involvement, how to embed this approach within a wider research study, and the broader social and communication skills needed to collaborate with diverse communities.

Alongside researchers it is just as important to equip the community with tools that enable them to have equal benefits in the process. The potential benefits for communities are wide-ranging but may include the ability to influence research and the changes it brings about, developing new skills (e.g. research, communication, subject knowledge), or forming new partnerships. What is perhaps most important is that community members have the opportunity to share which benefits they hope to gain from their involvement (both at the outset and as a research project progresses), and that community involvement actively seeks to enable these benefits.

Particularly young people should be provided with the skills and knowledge to be advocates of co-creation in Prevention Science. Many of the methods and concepts which underpin Prevention Science are critical skills which young people are likely to need as they navigate the world of work and wider society. For instance, how can they be supported to critically evaluate information (social media, news stories) about key social problems? How do we equip them with the skills to work in a world in which data and digital tools are increasingly central? Or address complex problems such as climate change that require interdisciplinary collaboration? Which skills are needed for co-creation and collaboration? The skills which community members need for involvement in research are therefore transferable across many other aspects of their lives. There are opportunities to promote the acquisition of these skills within the education curriculum in ways which embed principles of Prevention Science, and to promote schools as health promoting institutions^1^. Young people themselves need to be involved in these discussions so that the benefits which they identify as being important are given the attention they deserve.

The example of embedding Prevention within schools’ curricula opens out to a broader issue about the distinction between Prevention *with* and Prevention *in* communities – a point helpfully raised by one of the peer reviewers of this paper. We acknowledge that our insights are situated and partial and are inevitably shaped by our own position as academic researchers. Whilst each of us live and work within communities, our work often involves taking research ideas to new communities to partner with them. Prevention Science with communities is one part of the broader field of Prevention in communities. Though we cannot explore it in detail here, we acknowledge that building skills and capacities for Prevention encompasses a diverse range of roles and experiences. To summarise the helpful points of the reviewer, we need to think about who is responsible for Prevention in communities. What level of understanding do communities have about how to identify and respond to risk and protective factors? How do communities choose from the many interventions which are promoted, so as to select those which are most likely to be effective and suited to their particular context? Do resources and networks exist to support this work? Might broader organisational and cultural norms need to shift to support an evidence-based approach which promotes Prevention over and above naïve individualistic strategies which are unlikely to be effective? Our response to this set of questions is that Prevention is a collective responsibility, and forming long term partnerships within communities, and between communities and researchers may be the best starting point.

The first studies where researchers co-created an intervention with relevant stakeholders are clear on one thing - it takes time to do this well. This means that you need to plan co-creation in advance and really think it through in joint development with the stakeholders. Moreover, funders should support the allocation of some funding to the co-creation phase, and without this being at the expense of the total amount available. Only by dedicating funds to the co-creation phase, can we ensure the proper involvement of stakeholders in Prevention. In addition to funding within specific projects, it is important that there are infrastructures (methodological specialists, trainers, ways of sharing good practice) that can push the development of this work forward and help legitimise its value. We can draw parallels here with the excellent work undertaken by the EUSPR and others around training of practitioners, which extends beyond skill development (critical though that is) to create systems which value and support individuals to work in new ways. This does not mean disregarding individuals’ past experiences and lessons learned. For researchers, conducting a thorough literature review is crucial to build on existing knowledge, avoiding the unnecessary effort of rediscovering previous work on a particular issue, population, or context. For stakeholders, the goal is to avoid ‘reinventing the wheel’ or wasting time by starting from scratch when existing tools could be refined and optimized. These methodological considerations can be reinforced through various types of training which facilitate effective interdisciplinary collaboration and ensure that both past insights and innovative solutions are fully utilized.

### Integrating Different Kinds of Evidence, and Maintaining the Principles of Evidence-Based Practice

A second challenge relates to the balance between the involvement of the community and safeguarding evidence-based-practice principles (EBP). Prevention Science draws on evidence and theories when designing interventions to maximise their effectiveness in acting on known risk and protective factors. It encompasses a commitment to delivery of interventions as planned (with fidelity) to retain their core elements.

Underlying community involvement is an assumption that it can achieve better fit between interventions and the needs of communities in which they are delivered. Herein, the debate revolves around concerns that interventions are designed so that a set of pre-specified activities generate change mechanisms, leading to hypothesized intervention outcomes. Where interventions demonstrate effectiveness, developers may be wary of - or prohibit, implementers from making changes to the content. It might be argued that modifying an effective intervention (by adding, removing, or altering activities) could jeopardize its effectiveness. Conversely, the core principle of community-involvement is that adaptations may be necessary for interventions to better meet the needs (and achieve their goals) within specific settings. The community might wish to take a divergent path altogether (address different priorities or adopt another intervention). While academics are trained to establish a standardized framework for implementation, aiming to minimize variances that could bias the impact and results, stakeholders often excel in adapting interventions to the specific audience they serve. Collaboration between these two ‘worlds’ should be valued for how it draws together complementary strengths. Researchers must find ways to evaluate interventions at a macro level, while stakeholders need to be mindful of the unique factors introduced by each intervention, whether due to their own actions or the context in which the intervention takes place. Together, such partnerships ensure rigor and relevance in addressing complex issues.

This tension was framed nicely in Professor John Toumbourou’s conference keynote, who described ‘top-down’ and ‘bottom-up’ approaches, and the risks of neglecting what we know about ‘what works’ (and doesn’t) if we focus only on a ‘bottom-up’ approach. The balance between community-involvement and retaining evidence-based principles has been debated for many years. A recent systematic review of youth involvement in alcohol misuse prevention (Aresi et al., 2023) argued that the growth of participatory approaches has been driven both by ‘rights-based’ and ‘empirical’, perspectives, which are sometimes in tension. For example, the authors suggest that research driven by a rights-based approach extends significant agency to young people but does not always progress to intervention implementation or evaluation. Conversely, ‘research-based interventions’, whilst rigorously evaluated may fail to take full account of the broader community context and sometimes restrict the extent to which young people can meaningfully influence the process.

Researchers have suggested that some flexibility is required to support implementation - e.g. intervention transferability into different contexts, provided that the core elements of the programme are maintained, and that the adaptations made are clearly understood (Moore et al., 2021); see also Dane and Schneider (1998). This aligns with a realist approach, which conceptualizes interventions as sets of resources that, when introduced into a context, generate mechanisms leading to outcomes (Bonell et al., 2016). Contexts are best thought of as active systems which interact in complex ways with interventions (Greenhalgh & Manzano, 2022). Realist approaches offer valuable insights into how we might develop and adapt interventions for different contexts whilst retaining their intended outcomes, and in ways which optimize their sustainability. Interventions might therefore be seen as ‘events in systems’ - they may be tailored to, and interact with, existing aspects of systems, sometimes varying in their exact form, whilst seeking to maintain a common function (Hawe et al., 2009). For such interventions, there are challenges for evaluators in understanding which aspects of implementation need to be described, and how to determine if tailoring of forms remains faithful to intervention function.

In the final plenary round table discussion, Glenn Laverack described a ‘third way’ - the ‘middle’ - to navigate the tensions between ‘top-down’ and ‘bottom-up’ approaches. Community involvement need not be in opposition to research evidence or the principles of EBP. The evidence base – and the researchers who work with it, bring vital knowledge about intervention development and evaluation. We know a great deal about successful prevention strategies (and the mechanisms through which they operate), approaches which are harmful, and the complexities of how interventions interact with local contexts (hence the many papers in the conference on implementation processes). Community involvement sits within these principles and frameworks. One only needs to look at intervention systems such as Communities that Care to see this approach at work with great success.

Community involvement has the potential to strengthen our work through helping us maximise the relevance, value, and acceptability of interventions for those who receive them. It can offer potential solutions to the critical challenges which we face, such as participant recruitment and retention, optimising data collection procedures, and the best ways to share research results with participants and the wider public. When we work to prioritise problems which require new/adapted interventions, community involvement provides an important voice. The same points apply when developing a new intervention - which needs to have meaning and acceptability amongst the target population. As discussed at the conference, community involvement does not mean simplistically asking people what we should do to address, say alcohol misuse, or whether they like an intervention - a point touched upon during John Toumbourou’s presentation. Similarly, the need to retain core elements of interventions was discussed in pre-conference workshops and main sessions. An example of what good community involvement might look like is offered by the Communities that Care (CTC) system (Toumbourou et al., 2019). It is underpinned by a strong conceptual framework, and places significant emphasis on training and coalition building. Data on known risk and protective factors (e.g. for alcohol use) is used by communities to identify priorities for action, with guidance also offered on evidence-based interventions which may best act upon these factors. Thus, CTC enables communities to adopt an evidence-based approach which is simultaneously tailored to their specific needs and context (Toumbourou et al., 2019).

The goal is to develop effective interventions, and to achieve this we need to integrate different kinds of evidence. Interventions should be informed by research evidence on mechanisms and activities known to be efficacious (e.g. aspects of family attachment/bonding). But such strategies will only be truly effective if they ‘fit’ the contexts into which they are introduced, can reach those they aim to serve, and have meaning and acceptability. Community involvement therefore involves bringing together the lived experiences of communities with research evidence and asking the right kinds of questions. When researchers partner with communities, they might pose questions that help identify priorities for action (‘What are the key public health issues in this community, and for the different groups within it?). This helps to create context and specificity. Once a problem has been articulated, researcher-community partnerships can think about which risk and protective factors might best address it (utilising the research evidence) whilst drawing on community perspectives about how such research can be applied in their setting. This is not to understate the potential complexities of articulating what is important, including how different groups may bring contrasting or conflicting priorities. In their conference campfire session, Jo White and colleagues described how the introduction of low traffic neighbourhoods (LTNs) in Bristol, England - with the aim of improving physical and mental health, faced significant opposition and ‘polarised’ debates, including from the media. They highlighted the importance of community involvement starting early in the process, and the value of using health evidence (e.g. the link between air pollution and respiratory conditions) to frame changes in the organisation of urban spaces (and not a desire to restrict the freedom of individual to travel by motor vehicle) (UWE Bristol, 2024).

### Assessing Community Involvement – Defining Quality?

Challenges relating to skills/capacity and the integration of different kinds of evidence require the development of a body of knowledge on methods for community involvement, its reporting and assessment. Through this endeavour we can fully realise the potential of community involvement. We need to ask what good community involvement looks like, and how we know when we see it. A starting point is that it should generate mutual benefit. It helps strengthen the quality and impact of research, whilst also bringing benefits for the members of the public– it is scientifically rigorous whilst drawing actively on community perspectives and priorities.

What is considered beneficial for one group may not hold the same value for another. Stakeholders and recipients of interventions should also consider what constitutes ‘good’ academic involvement and critically assess it. After all, should the benefits of an intervention be carefully analysed and understood by the recipients, allowing them to make sense of it? Or should these benefits be simply embraced without further scrutiny? Where priorities for new interventions or research are generated with little or no involvement of the communities who may later be asked to contribute to their implementation, there are many problematic outcomes which may result. Perhaps the chosen problem or intervention / target is not seen as important within the community, and fit with local context is thus poor. Or, even worse, the intervention topic *is* seen as important (in a general sense), but the community does not consider there to be a significant problem to address prior to the issue being raised by a research study (for instance), with potential for labelling and negative associations to be created.

To achieve the goal of good community involvement, do we need to generate a shared understanding of its principles within Prevention Science? Or does this already exist, albeit in sometimes disconnected pockets of expertise? How do we design community involvement within our work? What needs to be reported within our publications to ensure that we learn from successes and challenges? How do we know whether community involvement has made a difference (to research, to the practice of researchers, or to the community)? Returning to the potential benefits for community members which we highlight above, it is critical to understand whether these have been realised, and if not, why not. Community involvement that helps to progress the completion of a research project, but which does not bring benefits to community members raises serious ethical concerns. If we understand the mechanisms through which community involvement generates benefits, we can build a science that informs future work. Such knowledge should not be siloed – it needs to be integrated within our broader methodological guidance and standards. This will help to embed community involvement within research studies and help us find the ‘middle.’

As with all our work, we need to avoid doing harm when working with communities. Such harms might include raising unfulfilled expectations that we will utilise community insights; ‘parachuting’ into communities and seeking input without an ongoing dialogue to explain how it has informed the research process; or asking people in broad and vague ways about ‘what to do’ but without reference to what we already know regarding effective approaches, and then developing potentially harmful responses. It is important to ask ourselves (and to be challenged by others) as to whether our community involvement is genuine (i.e. have key decisions already been made?) or tokenistic (it is unlikely to have meaningful influence). Tokenistic or inauthentic community involvement demonstrates a lack of respect and has clear potential to generate poor scientific conclusions. It also risks undermining the public’s trust in researchers (in general) and science – a precious commodity which we need to earn and maintain. Whilst ‘parachuting’ in is problematic, we also need to avoid the potential harms of ‘parachuting’ out. The short-term benefits of community involvement within time limited research projects may be undone if researchers leave abruptly. Relationships are built over time, and long-term partnerships (for which funding is unfortunately often scarce) can avoid the negative impacts of multiple and disjointed project specific requests for input. Hence the value of infrastructures to build capacity for community involvement and help ensure long term sustainability.

Finally, we must always consider carefully issues of inclusion (Who are we talking to, who is not included?), power dynamics (Aresi et al., 2023) (who gets to speak and for whom) and any negative consequences which may follow from community involvement (e.g. where individuals criticise existing policies, etc.). Where power balances are not addressed, there is clear potential for community involvement in research to dis-empower certain groups or individuals – an iatrogenic effect which is the antithesis of what we are aiming to achieve.

### Where Next?

We have reflected on the key insights gained from the 2024 EUSPR conference. They related to three important dimensions of community involvement within Prevention Science – the skills and training needed, how it might be integrated within our scientific work, and how we define and assess quality and impact.

These are complex challenges, spanning capacity building, methodological innovation and development of new language and frameworks. But Prevention Science is well placed to expand community involvement, given its commitment to and expertise in methodological innovation, grounding scientific rigour in research that takes place within communities, and its embodiment of working across boundaries. It has a long track record in developing standards and guidelines, and systems for critically evaluating research quality and impact (e.g. the Xchange registry). Many examples of community involvement (and other forms of public involvement) already exist within Prevention Science, with much to build on and to learn from – we are not starting from the beginning. In the broader field of public involvement (including public health research) there is a wealth of published work and methodological guidance which we can draw on and adapt to ensure it meets our needs.

The EUSPR’s annual conference is peripatetic, engaging with local prevention systems across European nations, seeking to understand the needs of communities in diverse contexts. Thus, in Cremona, we explored the principles of Prevention in and with communities, and how they play out in the Lombardy region of Italy, whilst also exchanging insights from many other countries.

To answer the question ‘Where next?’ we suggest some specific steps. Through the annual conference and other activities of the EUSPR (and international SPRs) we have an ideal opportunity to address researchers’ training needs. Face-to-face workshops, online training sessions (which have already happened) can build skills and confidence in community involvement. We hope that this commentary will be a starting point for others to discuss how they envisage community involvement in Prevention Science can develop in the future. If we are to successfully integrate community involvement within Prevention Science, we will need to develop a shared understanding of what good practice looks like. Our existing methodological standards, guidelines and evaluation frameworks may need revisiting. When assessing research quality, we need to understand if the work in question has involved the communities it is designed to impact, and how their insights have been used to strengthen it. This can only happen if research publications make explicit whether community involvement took place, the methods employed and how this shaped the design and evaluation of interventions.

Finally, there are untapped opportunities to involve members of the public in EUSPR conferences and bring the voice of communities into our exchange of knowledge and ideas. What might the young people of Cremona, Berlin or Sarajevo have to say to us about the complex topics we grapple with, such as addressing climate change, engaging AI, or embedding prevention interventions within schools? What might we have to share with them? As the EUSPR celebrated its fifteenth birthday in Cremona it was looking back - seeing how far it has come and looking forward to what the society and Prevention Science might be like 15 years from now. Doing Prevention in and with communities has much to offer the future development of our field and where we go next, building on the lessons learned thus far, and the insights we gained in Cremona.

## Footnotes

1 In Wales, for instance, health and wellbeing is now a distinct ‘area of learning and Experience’ within the new national school curriculum - Health and Well-being: Introduction - Hwb (gov.wales).
